# Preferences for HIV test characteristics among young, Black Men Who Have Sex With Men (MSM) and transgender women: Implications for consistent HIV testing

**DOI:** 10.1371/journal.pone.0192936

**Published:** 2018-02-20

**Authors:** Victoria Frye, Leo Wilton, Sabina Hirshfield, Mary Ann Chiasson, Debbie Lucy, DaShawn Usher, Jermaine McCrossin, Emily Greene, Beryl Koblin, for the All About Me Study Team

**Affiliations:** 1 Department of Community Health and Social Medicine, Sophie Davis School of Biomedical Education, CUNY School of Medicine, City University of New York, New York, New York, United States of America; 2 Laboratory of Social and Behavioral Sciences, New York Blood Center, New York, New York, United States of America; 3 Department of Human Development, College of Community and Public Affairs (CCPA), Binghamton University, Binghamton, New York, United States of America; 4 Faculty of Humanities, University of Johannesburg, Johannesburg, South Africa; 5 Division of Research and Evaluation, Public Health Solutions, New York, New York, United States of America; 6 Project ACHIEVE, Laboratory of Infectious Disease Prevention, New York Blood Center, New York, New York, United States of America; 7 Department of Epidemiology, Columbia University Mailman School of Public Health, New York, New York, United States of America; University of Queensland, AUSTRALIA

## Abstract

**Background:**

Promoting consistent HIV testing is critical among young, Black Men Who Have Sex With Men (MSM) and transgender women who are overrepresented among new HIV cases in the United States. New HIV test options are available, including mobile unit testing, one-minute testing, at home or self-testing and couples HIV testing and counseling (CHTC). In the context of these newer options, the objective of this study was to explore whether and how preferences for specific characteristics of the tests acted as barriers to and/or facilitators of testing in general and consistent testing specifically among young Black MSM and transgender women aged 16 to 29.

**Methods:**

We conducted 30 qualitative, semi-structured, in-depth interviews with young, Black, gay, bisexual or MSM and transgender women in the New York City metropolitan area to identify preferences for specific HIV tests and aspects of HIV testing options. Participants were primarily recruited from online and mobile sites, followed by community-based, face-to-face recruitment strategies to specifically reach younger participants. Thematic coding was utilized to analyze the qualitative data based on a grounded theoretical approach.

**Results:**

We identified how past experiences, perceived test characteristics (e.g., accuracy, cost, etc.) and beliefs about the “fit” between the individual, and the test relate to preferred testing methods and consistent testing. Three major themes emerged as important to preferences for HIV testing methods: the perceived accuracy of the test method, venue characteristics, and lack of knowledge or experience with the newer testing options, including self-testing and CHTC.

**Conclusions:**

These findings suggest that increasing awareness of and access to newer HIV testing options (e.g., free or reduced price on home or self-tests or CHTC available at all testing venues) is critical if these new options are to facilitate increased levels of consistent testing among young, Black MSM and transgender women. Addressing perceptions of test accuracy and supporting front line staff in creating welcoming and safe testing environments may be key intervention targets. Connecting young Black MSM and transgender women to the best test option, given preferences for specific characteristics, may support more and more consistent HIV testing.

## Background

New HIV infections among gay, bisexual and other men who have sex with men (MSM) ages 13 to 24 increased by 133% between 2001 and 2011 in the United States (US) (Johnson et al., 2014) [1]. HIV prevalence and incidence are disproportionately high among young, Black MSM and transgender women [2–7]. Recent research has observed a 5.9% annual HIV incidence among young, Black MSM and transgender women aged 18 to 30 in six major urban areas in the US [4]. New York City (NYC) has the largest number of newly diagnosed HIV infections among MSM in the US [8]. Between 2001 and 2010 in NYC, new HIV diagnoses increased among young MSM, with about half of all new cases among young, Black MSM [9]. Recent NYC surveillance data have revealed declines in new infections among MSM, but they have not been statistically significant; in the first half of 2013 MSM made up 60% of all new infections in NYC [10]. Further, while studies on Black transgender women have been limited, recent epidemiologic data has shown that approximately 90% of newly HIV diagnosed transgender women in NYC were among young, Black and Latina transgender women [11].

HIV testing is the entrance point to a range of HIV prevention and treatment options, such as pre-exposure prophylaxis (PrEP) and ART (antiretroviral therapy) treatment, and is therefore a critical public health tool to help contain the HIV epidemic in the US [12–20]. It is crucial that individuals learn of HIV infection as immediately as possible, as those who are unaware of their HIV infection are more likely to transmit HIV to others due to higher viral loads and higher prevalence of sexual HIV transmission risk behaviors [2, 8, 21–22]. Testing offers individuals who engage in high risk sex and receive a negative test result the opportunity to consider or adopt PrEP, a highly effective, biomedical HIV prevention method, learn about PEP and/or work towards other risk reduction strategies [12–13, 23–25]. For these reasons, the Centers for Disease Control and Prevention (CDC) has recommended that MSM and transgender women who engage in certain risk behaviors test consistently or every 3–6 months [8, 20, 25–26].

Young Black MSM and transgender women are more likely to be unaware of their HIV infection [8, 27–29]. In the 2014 National Health Behavior Survey (NHBS), 25.0% of Black MSM (aged 18–24) and 23.0% (aged 25–29) had not tested in the prior 12 months; [8] further, HIV testing is less frequent among undiagnosed HIV-infected Black MSM compared to HIV-uninfected Black men [16]. The Brothers Study (HPTN [HIV Prevention Trials Network] 061) found that almost a quarter of the 1,301 Black MSM with no prior HIV diagnosis had not tested in the past year and MSM who had not tested recently were four times more likely to have an undiagnosed HIV infection compared to those who had tested recently [22]. Thus, consistent testing among this population subgroup is a critical public health goal.

Barriers to HIV testing among Black MSM and transgender women include fear, stigma, disclosure of sexual/gender identity, lack of anonymity, concerns about confidentiality, discomfort talking to a counselor and concerns about the accuracy of rapid tests [30–34]. Facilitators of testing include visits to health providers, perceived risk (a partner has HIV), sexually transmitted infections (STIs), experiencing STI or acute HIV infection symptoms, condom slips, being told by a partner to get tested, and social support [35–36]. One of the few studies focused exclusively on young Black MSM found that the factors most strongly associated with a lifetime history of HIV testing were knowing a comfortable place to test and social support to discuss HIV and sexuality issues [37]. Peer social support has been associated with recent HIV testing among young Black gay, bisexual, and other men who have sex with men, as has social support in community samples of young Black MSM and transgender women [38–41]. There is limited research on what predicts *consistent* HIV testing among young Black MSM and transgender women in an era of increased test options.

The emergence in recent years of several new testing methods and technologies have increased HIV testing options, which may contribute to the goal of increasing consistent testing among higher risk groups. These newer methods address some of the established barriers to HIV testing described above. For example, home-based or self-testing uses a 20-minute, oral fluid-based technology and allows individuals to operate the test and receive results privately and at their convenience, alleviating the burden of having to access traditional testing venues or a health care provider, which may elicit stigma and confidentiality concerns [30, 42–44]. In addition, couples HIV testing and counseling (CHTC) applies traditional rapid testing methods in the context of a counselor-delivered session with two or more individuals who consider themselves to be a couple or have sex together and seek testing services together. This approach is delivered by trained test counselors and involves individuals receiving test results together, supporting each other in the context of the results received and planning prospectively for risk reduction via discussion of sexual agreements [45]. In addition to these new operator (self), venue (home) and counseling (couple) options, traditional HIV testing with a counselor at a fixed site or on a mobile unit is still widely available, typically using either the 20-minute oral fluid- or finger stick/blood-based rapid test. Finally, the one-minute HIV test uses a finger stick blood specimen and offers results in one minute.

In the context of these newer options and the need for consistent HIV testing among high risk MSM, we explored whether and how preferences for specific characteristics of the tests acted as barriers to and/or facilitators of testing in general and consistent testing specifically among young Black MSM and transgender women aged 16 to 29. We also evaluated perceptions of the “fit” between available methods and their characteristics and participants’ past and future testing needs. The findings presented here result from a formative research phase of a larger study designed to inform the development of a communication technology-based intervention to increase consistent HIV testing among this age group.

## Methods

### Recruitment and eligibility

Participants were recruited between February and May 2014 through online and mobile applications (apps) used by young, Black MSM and transgender women in the New York City metropolitan area (e.g., Craigslist, Black Gay Chat [BGC], Facebook, etc.) and the homepage of a local organization for gay men of African descent. Face-to-face recruitment was used to reach the participants aged 16 to 19. Recruitment language and ads explicitly included transgender women. Using these methods, 396 individuals (MSM, N = 378; transgender women, N = 18) completed the online screener and 78 (20%) were eligible. Face-to-face recruitment occurred at two events (a kiki ball or mini-House Ball, and at an outdoor venue, “The Piers”, in lower Manhattan) where young, Black MSM and transgender women often gather; 18 contacts were collected and 14 were eligible (78%).

To be eligible for the study participants had to: (1) report being male at birth; (2) self-identify as Black, African American, Caribbean Black, African Black or multiethnic Black; (3) be able to read and respond in English; (4) be between 16–29 years of age; (5) not be known to be HIV-infected; (6) report insertive or receptive anal intercourse with a man or transgender woman in the last 12 months; (7) reside in the NYC metropolitan area; and (8) provide informed consent or assent for the study. Participants who were enrolled in any other HIV research study involving HIV testing or who had been a participant in an HIV vaccine trial were not eligible. Sexual identity was not a criterion, as self-reported behavior was used to establish eligibility. To provide appropriate contrasts, we attempted to interview participants who had and had not tested for HIV at least once in the last six months. The recruitment methods resulted in a sample of 30 participants, which was the target sample for the study and where we reached saturation with regard to the themes emerging around preferences for, barriers to and facilitators of HIV testing in the context of newer options.

### Study procedures

Participants who screened as preliminarily eligible were contacted by telephone to confirm eligibility and set up a study visit appointment. All participants provided written informed consent or assent and the study was reviewed and approved by the Institutional Review Boards (IRBs) of all participating institutions (New York Blood Center, Binghamton University, and Public Health Solutions). Eligible participants under the age of 18 years were considered mature minors by the IRBs and thus provided written informed assent. After providing information about the study and prior to obtaining assent, the minor participant was introduced to and met with the minor advocate. The advocate was a staff member who was not involved in the assent or this study’s procedures. All information provided by the participant during the meeting with the advocate was confidential and not shared with any other staff members. At the end of the study visit, the participant was provided the opportunity to meet with the advocate again and given the advocate’s contact information for any future questions. Eligible participants completed a 60-minute, qualitative, semi-structured, in-depth interview and a brief web-based survey on demographics, health characteristics, and sexual behavior. Participants who completed the study visit received $30 compensation for their time, along with a two-way Metrocard for travel. Interviews were conducted by trained and experienced interviewers in a private room and were audio-recorded and professionally transcribed. All interviews were checked for accuracy by the primary qualitative study co-investigators (VF and LW).

### Interview guide

The in-depth interview was semi-structured, employing a guide that explored a variety of issues including: personal background; experience of and connection to communities; thoughts and feelings about HIV testing and most recent testing experience; experiences with and perceived facilitators of and barriers to newer testing methods (e.g., couples testing, also known as “Testing Together”), venues (e.g., mobile units), and operators (e.g., self-testing); and use of and thoughts about web- and smart phone app-based technologies for health and testing purposes. The interview guide was based on a conceptual model that identified factors at four levels, the test level, the individual level, the situation level, and the socio-contextual level. At the test level, we asked questions designed to elicit accounts of the influence of test characteristics, such as availability, cost, fluid used/specimen collected, test operator, familiarity, complexity, time for results, and privacy/confidentiality on testing and test preference. At the individual level, we explored the influence of fear, anxiety, stigma, past experiences and “type” of tester, [46] as well as medical mistrust. In terms of the situation and social context, we assessed how cues to action, situational access, counseling type and quality, peer norms, and general social context related to testing. In this report, we focus on data related to whether and how specific test characteristics connect to preferences for, barriers to and facilitators of testing in general and consistent testing specifically. During the interview, interviewers described the two commercial self-tests currently available, the OraQuick In-Home HIV Test^®^ (OraSure Technologies; Bethlehem, Pennsylvania), approved in July 2012, and the Home Access^®^ HIV-1 Test System (Home Access Health Corporation; Hoffman Estates, Illinois), noting the cost of, process used, and specimen collected with each method.

### Analytic methods

Using a grounded theoretical approach, we applied both contextualizing and categorizing strategies to code and analyze the in-depth interview data [47–48]. Immediately after each interview, the interviewer developed a summary document that described the participant’s life history, experiences with HIV testing, dominant themes of the interview and selected social characteristics. All summaries and transcripts were read by the analytic team and the lead analysts (the first two authors). A list of analytic areas represented in the data were composed and coded. The lead analysts re-read all transcripts and conducted preliminary coding of the text, based on interview topics and themes that were identified through the reading of the transcripts. The lead analysts coded the first five interviews and inter-rater reliability was evaluated; although the reliability was acceptable, the analysts discussed the inconsistent codes and coded another five interviews. These were compared and inter-rater reliability was found to be acceptable; the remaining 20 interviews were coded by the second author in QSR International’s NVivo 10 qualitative software (QSR International Pty Ltd. Version 9, 2010). Once the data were coded, queries were run on the following codes: “HIV test and person type”, “HIV test availability, characteristics and past testing”, “HIV testing preferred method”, “HIV testing accuracy”, “HIV testing barriers-past”, “HIV testing facilitators—past”, as well as text coded according to specific characteristics of testing, such as “fluid type”, “operator”, and “venue”. These portions of text were compiled, read, and analyzed by the lead author. Finally, all transcripts were re-read in full to confirm that no reported quote was overly de-contextualized from the larger narrative. We paid attention to data that did not confirm emerging or dominant themes, noting these in the results. In addition, we assessed whether the dominant themes were evenly represented among the four participants who self-identified as transgender and/or described being gender fluid and whether they varied by recent (past six months) HIV testing history. All participant quotes are identified by initials (not actual) and selected sociodemographic characteristics to protect privacy.

## Results

### Sample

The mean age of the sample was 23.7 (SD = 3.4), with one 17-year old participant and eight participants who were between 18 and 21 years of age; 21 participants were aged 22 to 29. Forty percent reported residing in the Bronx, 30% in Brooklyn, 27% in Manhattan, and 3% in Queens. The majority (87%) self-identified as African-American, with 23% identifying as Caribbean or West Indian or 20% as Afro-Latino. Ninety-three percent of participants self-identified as male, with two participants identifying on the computer-based survey as transgender male-to-female. During the interviews, however, another two participants revealed that they self-identified as gender fluid and sometimes identified as male-to-female transgender, sometimes as female and sometimes as male. Table 1 provides additional sociodemographic, perceived HIV risk and past HIV testing characteristics of the sample.

**Table 1:** Sociodemographic, health, HIV risk perception, and HIV testing characteristics of sample (N = 30^‡^).

Factor	N	Percent
**Age** (mean; SD)	23.7	3.4
Race/ethnicity*		
African-American	26	87%
Caribbean/West Indian	7	23%
Afro-Latino	6	20%
Other	1	3%
**Gender identity**		
Male	28	93%
Transgender (male to female)	2	3%
**Sexual orientation**		
Gay	21	70%
Bisexual	8	21%
Heterosexual/straight	1	3%
**Borough of residence**		
Bronx	12	40%
Brooklyn	9	30%
Manhattan	8	27%
Queens	1	3%
Staten Island	0	0%
**US-born** (N = 29)	27	89%
**Education** (N = 28)		
High school degree or GED	7	23%
Technical School	2	7%
Some college	15	50%
College degree or more	4	13%
**Income greater than $20,000 per year** (N = 28)	12	40%
Provision of rent, food, utilities and living expenses*		
**Self**	21	69%
Parents/other relatives	13	43%
Other	2	7%
**Income insecurity** (**very or fairly often)** (N = 28)	9	30%
**Employment** (N = 27)		
Full-time work	10	33%
Part-time work	6	20%
Not working	10	33%
Working off the books	1	3%
**Health insurance** (N = 28)	25	82%
**Medical care receipt (usual)** (N = 28)		
private MD	17	56%
emergency department	7	23%
community clinic	4	13%
**Perceived likelihood of getting HIV** (N = 28)		
Extremely or very unlikely	13	43%
Unlikely	11	36%
Likely	4	13%
Extremely or very likely	0	0%
**Worried about getting HIV** (N = 28)	17	56%
**HIV tested, lifetime**	29	96%
**HIV tested, past six months** (N = 28)	19	63%
**Venue most recent HIV test** (n = 27)		
Community health/free clinic or CBO	14	46%
Private medical doctor	7	23%
Mobile testing unit	2	7%
Emergency department, research study or other	4	13%
**Specimen type most recent HIV test** (N = 27)		
Whole blood draw	11	36%
Finger prick	6	20%
Oral fluid	10	33%

* totals to greater than 30, as multiple answer choices were accepted

^‡^ Ns for each factor are listed when one or more participant declined to answer

### Established barriers to and facilitators of HIV testing

We confirmed results of previous research that several factors continue to act as barriers to and facilitators of HIV testing in this population. These included: knowledge of and access to HIV testing sites and test methods, fear of a reactive HIV test result, HIV stigma, and concerns around confidentiality and privacy. In terms of access to newer testing methods, the majority of participants were unaware of the CHTC option (which was not widely available at the time of the interviews), although they were open to and enthusiastic about CHTC. One participant noted its usefulness in planning for safety with a future partner, saying “I would do the Testing Together to get to know the person and where they stand.” (NO5; 24, African-American, male, Bronx, not tested P6M) Another participant noted that if the new methods were widely disseminated, they would be used, stating “I think maybe I could be—not really comfortable with it, but I feel like if it was much more available, then I would possibly take advantage of it.” (ST1; 21, African-American, male, Bronx, tested P6M) A transgender participant said in response to being told of the various testing options that there were “a lot more options than what I am aware. I’m excited. I’m excited.” (WX5; 20, African-American, gender fluid, Brooklyn, not tested P6M)

Some participants noted that access to existing testing methods was still a problem, particularly in the Bronx, with too few fixed testing sites available. One participant said, “New York City has facilities specifically for that spread around the borough, but there’s only like two a borough.” (DE4; 20, African-American, male, Manhattan, not tested P6M) Related to access to the self-test, the cost of the test emerged as a barrier, consistent with preliminary reports in the literature and our analysis of these data (Frye et al., 2015). The majority of participants expressed anxiety or nervousness when asked their “first thoughts” on HIV testing, reinforcing that the testing experience continues to be fraught with anxiety for most young, Black MSM and transgender women. One participant said, “I’m high anxiety so, you know, that will definitely get my anxiety going and stuff, just yes, worrisome.” (CD3; 28, Afro-Latino, male, Bronx, not tested P6M) Another participant simply said “fear,” whereas another stated “sickness, depression, condoms, wrap it up, and depression.” (NO5; 24, African-American, male, Bronx, not tested P6M) One stated “It’s draining.” (XY6; 22, African-American, male, Bronx, tested P6M) The role of stigma associated with testing also emerged for several participants when asked about their first thoughts on testing; one participant said “I guess shame. Even though I work with the disease itself, I’m not scared of it. I respect it. But it still holds a stigma. You don’t necessarily want that stigma attached. … So it’s not a nice feeling.” (RS9; 29, African-American, male, Brooklyn, tested P6M) Finally, as in previous research, issues related to confidentiality emerged, with some participants perceiving fixed site waiting rooms and mobile units to be antithetical to privacy, for example.

### Emergent barriers to and facilitators of HIV testing

In this section, we present novel results describing how preferences for selected characteristics may constitute barriers to and facilitators of testing and explore how some of these preferences interacted with each other. We describe preferences that emerged from the data most strongly, organized by test characteristics (e.g., specimen type required, reported accuracy, etc.), individual/personal characteristics (e.g., perceived accuracy, anxiety, etc.), and the situational or contextual characteristics (e.g., venue, location, etc.).

### Test and individual characteristics

Related to the test characteristics, factors such as type of specimen collected, reported accuracy, familiarity with the actual testing process and speed of the test results emerged as important to test preferences. Several participants expressed a strong preference for blood-based tests and venipuncture due to greater accuracy of these tests and fewer false negative test results, as compared to oral fluid-based tests. Some participants believed that blood-based tests, venipuncture with a western blot test in particular, were more sensitive, resulting in shorter window periods and greater confidence in blood-based tests. For example, one participant said:

“But like I said, with me it’s not so much the convenience and stuff like that. Sometimes I just don’t trust the results for somebody to swab your mouth and a little thing of blood to detect. I mean—I know that this has been tested for ages before we even got it, so I know that there is, you know, they know what they’re doing, but it’s just me up in my head. … I prefer to get a needle in my arm and I’ll see you in a week to ten days.”

(BC2; 29, African-American, male, Bronx, not tested P6M)

Expressing greater confidence in blood-based rapid tests, another participant said, *“*For me, it’s more accurate. HIV lives in the blood. … Check the blood.” (DE4; 20, African-American, male, Manhattan, not tested P6M) Another said: “I really don’t trust it [the oral fluid], so every time I go in there and get it I think I don’t trust it.” (BC3; 19, African-American/Afro-Latino, male, Brooklyn, tested P6M) Another asked, “Isn’t the blood test more accurate? I really—I want to know. I want to be 100% sure. Every time I get tested, I want to be 100% sure that it’s correct—there’s no ands, ifs, or buts about it.” (FG6; 19, African-American, male, Brooklyn, tested P6M) One participant worried that the preference for blood-based tests may prevent some people from getting rapid HIV tests. This participant said: “I believe the only obstacle would be the fact of their preference of venipuncture over the rapid testing. I hope that doesn’t stop them from getting it. I hope they still think, you know, this is good enough for right now. Even though my preference is venipuncture, I hope they feel like I should do this right now just to at least know my status some way.” (DE4; 20, African-American, male, Manhattan, not tested P6M) This participant recognized the potential for the perception of significantly reduced accuracy of rapid HIV tests, oral fluid-based in particular, to act as a barrier to consistent or needed testing.

### Situational or contextual characteristics: Counselor and venue

In terms of situational or contextual characteristics, factors such as relationship status, past testing patterns, prior negative experiences with testing (e.g., specific counselors or providers and mobile units especially), counselor skill and compassion, and venue culture (e.g., courteousness, confidentiality, etc.) emerged as important to preferences for specific testing methods or test characteristics. Of special importance for several participants was the relationship they developed with their counselors and/or perceived attitudes of counselors towards the clients. One participant who sought testing every four months noted, “I mean it’s one of those—they’re there to do their jobs. So it’s not like they’re enthusiastic or they have enthusiasm to do the test for you. So it’s like a regular job for them.” (GH7; 25, African-American, male, Bronx, tested P6M) Counselor skill at taking a sample emerged as well in terms of wanting to return to either the venue and/or use the specimen collection method again. The same participant described an adverse oral fluid specimen collection experience where “the lady doing it—because they’re supposed to do it for you. So when they do it, it looks like they’re fighting with your mouth…and I’m like, ‘Is this really supposed to happen?’” (GH7; 25, African-American, male, Bronx, tested P6M) Another participant said that the attitude of the clinic workers to their roles was important noting:

“I went to one in [NYC neighborhood] and I really didn’t like it. [Why not?] I don’t know. The whole environment, the people that were there, and the workers, they weren’t—they sounded like they were pissed the whole day, so I was like, ‘This is not the place for me.’**…** The people [waiting to be seen], whatever they were doing, they looked upset as well. And they were on the phones arguing and stuff like that, so it was…”

(HI8; 26, African-American, male, Manhattan, tested P6M)

Other participants noted that selected testing venues, specifically mobile testing units, were not conducive to confidentiality, comfort, or confidence. For example, one participant who said that they would not seek a test on a mobile unit or van said:

“I don’t think I feel comfortable in vans. I just feel like vans are for delivering things that you need. I don’t think I should be getting my results while you’re parking or while you’re parked in the yellow line. It just doesn’t feel comfortable for me. It doesn’t feel like it’s real, honestly.”

(PQ7; 23, African-American, male, Manhattan, tested P6M)

The impermanence and mobility of the vans disconcerted some participants; when asked what disturbed them about the van one participant replied “Just the idea that they could just lock the doors and run off with you.” (FG6; 19, African-American, male, Brooklyn, tested P6M). Another noted, “I walk by them every single time. It’s—I don’t trust it.” (BC3; 19, African-American/Afro-Latino, male, Brooklyn, tested P6M) In contrast, a few participants appreciated the convenience and easy access that mobile testing units provide. One participant who was never tested said they would feel “less exposed” when testing on a van, suggesting that they did not want to be seen going into a fixed testing site (AB9; 20, African-American, male, Queens, never tested). Another participant noted that the vans often allowed testing with friends or had a positive energy (CD4; 17, Caribbean/West Indian, male, Bronx, not tested P6M).

### Testing method preferences over time

In addition to identifying characteristics of the tests and situation that drive preferences for testing methods, we asked participants about hypothetical preferences for various methods and test characteristics at different stages of their lives. Participants believed that they may benefit from a range of test options, noting that some methods would be good for them at different times in their lives. For example, a few participants felt that their current living situations would preclude their use of a self-test; for example, one participant noted “And the at-home test, my parents know that I’m sexually active and I identify as a homosexual, but I don’t think I would want to do it while they’re there…I don’t want them questioning anything like, “What are you doing?” I don’t want to have to go through those conversations.” (FG6; 19, African-American, male, Brooklyn, tested P6M)

In contrast, another participant imagined that they would combine self-testing at home (using oral fluids) with venipuncture at their health care provider’s office at a different stage in their life:

“Okay, the oral tests I would probably do every once in a while, at home, but, you know, to get the blood draw, I would probably do it every time I had my doctor’s visit. That would be the only times that I would change it up.”

(CD3; 28, Afro-Latino, male, Bronx, not tested P6M)

Another resident of the Bronx noted that having access to the self-test would allow them to test at home and would save them time during life stages characterized by time pressure and a hectic schedule (DE4; 20, African-American, male, Manhattan, not tested P6M).

Most participants indicated that CHTC (with a sex partner) would be of interest at time of life when they were partnered or “settled down,” although some said that the complexity of and commitment inherent in the method, specifically testing with a sex partner and discussion of sexual agreements, etc., would be a barrier. Further, the issue of trust while testing with a sex partner or friend emerged as a strong barrier to using this method at any life stage for several participants. With that said, a few participants reported that CHTC was an ideal way to test, particularly in the event of a reactive test result; one participant said this of having a friend or partner present:

“You have people to fall back on. You always want somebody to talk to after, you know, on the train ride home or something. People could calm you down. They could tell you things, they could tell you experiences, share stories.”

(QR8; 22, African-American, male, Brooklyn, tested P6M)

We explored with participants whether the existence of one of the options described, including the self-test, CHTC and the 1-minute test, would have increased the chance that participants would have tested at a time in their lives when they “thought they should have tested, but had not.” One participant noted that the 1-minute test would have made it more likely that they would have tested; another participant said they would have used the self-test to keep things private when they were younger and in need of testing. Similarly, another said that if the home test had been available when he was younger, he would have tested sooner. Another participant indicated that in the past if a testing option had been nearby, quick and convenient and he’d had a higher risk sexual experience, he would have been more likely to test at a time when a test was needed but not accessed. In terms of options that are missing from the current testing environment and that would potentially increase consistent testing, one participant lamented that results could still not be delivered by phone, which would reduce anxiety associated with receiving results. One participant noted that a more sensitive test would make them more likely to test more frequently, saying “if they said maybe it can be detected, I guess, in like a shorter time frame, then, I guess, that would maybe make me go every two or three months, if I was like really, really active.” (IJ9; 21, African-American, male, Manhattan, tested P6M) Most participants could not imagine another testing option that would significantly increase their chances of testing or testing consistently. One participant said:

“I’m not really sure that anything’s missing, because I think they’re all kind of convenient in a way. So, it’s like there’s not really a reason why you can’t go get yourself tested. They’re kind of available everywhere you go now at this point. So, it’s like there’s no excuse to not get tested.”

(CD4; 17, Caribbean/West Indian, male, Bronx, not tested P6M)

### Perceived person-method fit

Finally, when asked if “certain tests were a better fit for certain people”, most responses focused on use of the home or self-test. Numerous participants indicated that the self-test is useful to those who could afford the higher price, noting that they would gain the benefit and flexibility inherent in the test. A few participants stated with concern that self-testing may be preferred by a “certain type of person” (one participant described them as “secret closet people” (MN4; 29, African-American, transgender, Brooklyn, tested P6M), one who does not intend to disclose a positive result. They speculated that the type of person who uses a self-test may be afraid to be seen attending a clinic or asking for a test from their doctors. In terms of the preference for blood-based or venipuncture, one participant articulated what was described by several other participants: that the person who strongly prefers venipuncture is the person who “worries a lot, that needs to know and, you know, that wants to do it the perfect way the first time, that kind of a person.” (CD3; 28, Afro-Latino, male, Bronx, not tested P6M)

## Discussion

The numerous HIV testing options that are now available provide more choices to individuals at higher risk for HIV infection, for whom consistent HIV testing is recommended. However, there is limited understanding of how newer testing options are perceived, including what aspects of the new options are preferred, and how these preferences could increase the likelihood of testing and/or consistent testing [49–50]. In this study, we explored with young, Black MSM and transgender women their perceptions of various aspects of HIV testing methods in an effort to uncover whether selected test or testing experience characteristics, specifically test characteristics, individual preferences and situational or contextual factors, strongly drove preferences for specific testing methods. Three major themes emerged as important to preferences for HIV testing methods: the perceived accuracy of the test method, venue characteristics, and lack of knowledge or experience with the newer testing options, including self-testing and CHTC. This last finding suggests that dissemination of these options has been limited in this major metropolitan and high HIV prevalence area. There is a need for a concerted, city-led effort to disseminate self-testing and CHTC in the New York City area, with the goal of significantly increasing knowledge of and access to these newer text technologies.

A driver of preference for blood-based specimen collection was consistently described in terms of the greater perceived accuracy of blood as opposed to oral fluid sample-based tests. This preference was evident among both recent and not recent testers, suggesting that it acts as a barrier to testing for some but not all potential testers. It is likely that, despite the young age of the participants and thus relative lack of testing experience, the actual reduced sensitivity of oral fluid tests has been communicated to them and may be acting as a barrier to testing venues or methods that rely on oral fluids [8, 51]. In addition to the scholarly literature that documents the reduced sensitivity of oral fluid, the fact that OraQuick use resulted in a spate of false positives in the New York City area [52–53 may also be well-known among those people who test frequently and test counselors. As one participant noted, it is likely that this knowledge is acting as a barrier to testing or consistent testing, as it constitutes yet another source of anxiety for those who are in need of testing [27–34]. Most participants described fear and anxiety as the dominant emotional responses to the thought of HIV testing; that the test employed might not provide definitive information on HIV status adds to this baseline anxiety, and may cause some people to delay testing while searching for a blood-based option or postpone the test indefinitely. Because this analysis is based on qualitative data it is not possible to characterize how strong a barrier this factor is in delayed testing, but it may be an overlooked or underemphasized barrier that is exerting a stronger than expected effect on deferred HIV testing in the population subgroup.

Two related situational or contextual factors emerged as drivers of preference and desire to return for future testing: counselor interpersonal skills and venue culture. Several participants reported that they felt that HIV test counselors were only “doing a job” or “going through the motions,” suggesting that the importance and gravity of the testing process was not appreciated consistently by counselors and reflected in interactions with clients. This perception, based on actual experiences, may inhibit future consistent testing. Although recent research reported that the counseling component of voluntary counseling and testing (VCT) does not prevent incident STIs, [54–55] it continues to be an important part of comprehensive HIV prevention, as it allows clients to be evaluated for PrEP and other biomedical prevention approaches, such as PEP. It is important to gain a better understanding of how members of high-risk groups or populations, where medical mistrust is well-founded, [56–57] experience HIV test and PrEP/PEP counseling given the results presented here on the importance of counselor skills and venue culture.

Two additional findings emerged that have implications for HIV prevention and social services. First, the young people sampled here were able to imagine using a range of test approaches at various points in their lives, based on where they were in their lives. Although some participants noted that reluctance to test was unconnected to access and knowledge, and rather reflected fear of HIV, several stated that had the self-test been available to them when they were younger they would have used it. Similarly, the lack of easy access to all the testing approaches was perceived as a barrier to testing for very young, emerging adults. This strongly suggests that in addition to efforts to increase knowledge of and access to the full range of testing approaches, the public school system should integrate information on all testing approaches currently available as well as provide access to HIV testing in high schools and provide HIV self-tests to students who seek them [58]. Second, the finding that participants thought that a particular “type” of person might prefer to use a self-test is somewhat concerning. This suggests that that these tests could become stigmatized by association with perceived higher-risk or “closeted” individuals who do not intend to disclose a positive status. We noted that the self-test was identified by those who had not tested recently as an attractive option that they would use in the future and/or would have used previously to test. It is important that young people consider the self-test as one option among several that allows optimal sexual health management rather than a tool to maintain the fear, shame, and secrecy that some feel around HIV testing. With increased dissemination and distribution, possibly via public high schools, the self-test has a greater chance of being seen as a positive and accepted component of a consistent testing plan, capitalizing on the full range of available methods, that can flexibly meet different testing-related needs at various times of life or situations.

### Limitations

There are several limitations that deserve mention. First, despite an effort to focus on younger people, we interviewed just one person younger than age 18 and thus our findings derive primarily from a young adult population. Along these same lines, just four participants self-identified as either transgender (male to female) or gender fluid. Given the significant heterogeneity within the transgender population, for example preferred gender identity, pre-operative or post-operative status, health care history, we cannot draw strong conclusions related to the communities and subpopulations within the transgender population. In addition, we recruited participants primarily using web-based approaches, supplemented by in-person recruitment efforts to reach younger participants. Thus, the sample may over-represent individuals who gather on-line, as opposed to people who gather face-to-face at venues. This sampling strategy was, however, designed to reach young MSM and transgender women who would be likely to encounter our web-based testing intervention. Related to sampling, the results cannot be generalized to the larger population of young, Black MSM and transgender women; rather, these qualitative analyses are meant to generate novel findings around preferences for specific test characteristics in an era of increased testing options. Finally, most of our sample had tested for HIV within the last six months and few had direct experience with the testing options that we explored in the interviews. As CHTC and self-testing options are scaled up and formally disseminated, it will be important to conduct ongoing research into how these options are experienced by the groups at highest risk and most in need of testing options. If added options continue to be perceived as options for other people or other times of life, they will do little to increase the consistent testing that is required to trigger clinical evaluation for PrEP.

## Conclusions

These findings suggest that increasing awareness of and access to newer HIV testing options (e.g., free or reduced price on home tests or CHTC available at all testing venues), among young, Black MSM and transgender women is necessary if these new options are to facilitate increased levels of consistent testing in these at-risk populations. Effective dissemination of the methods, leading to easy access and uptake, is critical to this goal [49]. Further, as different methods of HIV testing may be preferred at different phases of life, the increased number of options may collectively act to increase chances of consistent testing across the life course [59]. Further experimental research would be needed to conclude if programs that help individuals develop tailored testing plans that take into account the full range of testing options and life stage increase consistent testing. Finally, HIV screening program planners and school health professionals should consider generating awareness and demand for the full range of test options through social and other media awareness-raising campaigns and provide easy access to all testing options [58].

## Supporting information

S1 File FINAL AAM in-depth interview guide. (DOC)
