# Supplementary material for: Preferences for HIV test characteristics among young, Black Men Who Have Sex With Men (MSM) and transgender women: Implications for consistent HIV testing
Source: PLoS One. 2018 Feb 20;13(2):e0192936. doi: 10.1371/journal.pone.0192936 (PMC5819791; doi:10.1371/journal.pone.0192936)
Supplement: S1 File — (DOC) [file pone.0192936.s001.doc]

**IN-DEPTH Interview Guide**

**Introduction**

“Thank you for deciding to be a part of this study. The purpose of this interview is to better understand your thoughts, opinions, and experiences with HIV testing and prevention. We will talk about your community experiences. We also will discuss some experiences that you’ve had related to sex. We hope that the information learned from this study will help us to better understand the health needs of Black men and transwomen who have sex with men or trans women.”

“There are several different ways that someone can get an HIV test these days. You can go to a clinic or community organization, you can tested on a van, you can test at your private doctor’s office, you can test at a clinic with a partner using “couples testing,” or you can do a self-test at home. This study is will be designing a brief Internet-based intervention for young Black men and transwomen who have sex with other men that will give them a personalized recommendation of an HIV testing option. It will be designed to be used with mobile phones. Your input will help us to create this intervention.”

“Please keep in mind that these questions do not have correct answers. We are really interested in your thoughts, experiences, and opinions. You do not have to answer any question that you do not wish to answer.”

“As a reminder, the information that you share during this interview will be treated confidentially. There are only two exceptions to confidentiality: If we learn of harm to a child, we are required by law to report it. And if you tell us you intend to harm or hurt yourself or another person we are required to report this to the proper authorities.”

“This interview will be digitally audio-recorded. The recording is made so that project staff can later carefully review what was talked about in the interview. The digital audio recording will be used to make a written transcript of the interview. The digital audio recording and transcripts will be labeled with a participant ID number only. Your name or any of the identifying information about yourself or anyone that you mention during the interview will not be associated with your responses.”

“Before we begin, would you tell me which gender pronoun you prefer that I use during the interview when referring to you? Also, when I turn on the tape, I’ll say the date and your participant identification number into the tape before we begin. I will also confirm that we have your consent to tape record the interview.“ Are there any questions before we begin?”

TURN ON TAPE:

The date is ____ /____ /_________.
This is interview # ________-________-________.

And for the record, I have your consent to tape record this interview?
YES/NO [If NO, turn off tape and continue with interview]
**The in-depth interview guide will provide a basis for discussion and does not have to be used verbatim.**

**Let’s begin with talking about your background.**

1. How did you hear about the study? What interested or motivated you to join the study?
2. How would you describe yourself?

**Probes:**

- Where did you grow up?
- Where do you live now? How long have you lived in this community? What have your experiences been like living in your community?
- What do you do with your time? Do you work? Go to school? Something else?
- How would describe yourself in terms of your identity or who you are as a person? Your racial, ethnic, or cultural identity? Your sexual identity? Your gender identity?
- Do you feel that you belong to any “community?” If so, which one(s)? What makes you feel connected to this community? What have been your experiences with the HIV community or people living with HIV and AIDS?

Now, I am going to ask you some questions about HIV testing.

1. Please tell me what comes to mind when you think about HIV testing.
2. Have you ever tested for HIV?
   1. If NO, if you wanted to get an HIV test, where would you go?
3. FOR THOSE WHO HAVE TESTED AND THOSE WHO HAVE NOT:

Have you ever thought that you ought to get tested for HIV, but did not?

- 1. What got in the way of you getting tested (that time)?
     1. PROBE FOR:
        1. Personal situation, relationship status, etc.
        2. Fear or anxiety about the test result
        3. Fear, anxiety or discomfort with testing (didn’t want to be seen, etc.)
        4. Feeling judged or negatively labeled based on being Black, same-sex behavior, being attracted to men, or doing something that has to do with HIV, like testing (HIV stigma and homophobia, but do not use these words)
        5. What family, friends, neighbors, may think
        6. Time and resources
        7. Access (inconvenient, don’t know where to go, etc.)
        8. Didn’t like organization or health care provider
        9. Didn’t want to know, wasn’t ready
        10. Would make you change: how you thought about yourself, your whole life, etc.
        11. Social norms/social support

1. FOR THOSE WHO HAVE TESTED:

Where did you get tested most recently? When was that?

- 1. What was the experience like? What did you like about it? What did you dislike?
     1. Probe for fit between issues below and testing location and type
  2. What made you get tested this most recent time? Did you plan it?
     1. PROBE FOR:
        1. Personal, situation, relationship status
        2. Triggering event, such as exposure, newly diagnosed STI or feeling unwell
        3. Fear, anxiety related to exposure
        4. Peace of mind
        5. Regular testing
        6. Easy access (convenient opportunity to test)
        7. New partner
        8. Feelings of responsibility
        9. Media message
        10. Other?

1. FOR THOSE WHO HAVE TESTED EVER:

Thinking about the time(s) in the past when you thought you should get tested, but did not, and thinking about this most recent test, what was different about the situations or yourself that allowed you get tested?

- - 1. PROBE FOR
       1. aspects of the test technology or setting
       2. facilitators and barriers

Now, I am going to talk a little bit about the new types of HIV tests that are available. You may know about these already or they may be new to you.

[Describe testing methods (i.e., rapid test, self-testing and couples counseling and testing) as well as venues for testing (e.g., clinics, community-based organizations, home)]

1. What do you think about each of these ways to test for HIV?
2. Which ones have you used?
3. Are there any that you have used that you would NOT use again? Why?
4. Are there any that you have used that you WOULD use again? Why?
5. What are the pros or good things about [insert each option]
6. What are the cons or not so good things about [insert each option]
7. Which ones do you prefer to use? Why?
8. Are there any ones that you have not used, that you could see using in
   the near future? Which one and why?
9. Was there a time in your life when the availability of a certain HIV testing method would have made it more likely that you would have tested?
   1. Probe for aspects of the test technology (i.e., length of time to wait, blood or oral, accuracy or sensitivity) that may have been important
10. Was there a time in your life when the availability of a certain HIV testing venue would have made it more likely that you would have tested?
    1. Probe for aspects of the test venue (i.e., proximity to home, staff, community based organization vs. medical care, home-based, etc.) that may have been important
11. Can you imagine a time in your life when you may want to use one of the methods that you have not already used? Which one and why?

PROBE FOR:

- 1. Role of venue
  2. Personal situation, relationship status, etc.
  3. Fear, anxiety or discomfort with testing method (didn’t want to be seen, etc.)
  4. Feeling judged or negatively labeled
  5. What family, friends, neighbors, may think
  6. Time and resources
  7. Access (inconvenient, don’t know where to go, etc.)
  8. Organization or health care provider
  9. Would make you change: how you thought about yourself, your whole life, etc.

1. What do you think would be most helpful or supportive for you in using one of these types of HIV tests?

PROBE FOR:

- 1. Role of venue
  2. Fear, anxiety or discomfort with testing method (didn’t want to be seen, etc.)
  3. Feeling judged or negatively labeled
  4. What family, friends, neighbors, may think
  5. Time and resources
  6. Access (inconvenient, don’t know where to go, etc.)
  7. Organization or health care provider
  8. Would make you change: how you thought about yourself, your whole life, etc.

1. What do you think are the things that may get in the way for you in using one of the types of HIV tests?

PROBE FOR:

- 1. Role of venue
  2. Lack of familiarity with method, complexity of method, etc.
  3. Personal situation, relationship status, etc.
  4. Fear, anxiety or discomfort with testing method (didn’t want to be seen, etc.)
  5. Feeling judged or negatively labeled
  6. What family, friends, neighbors, may think
  7. Time and resources
  8. Access (inconvenient, don’t know where to go, etc.)
  9. Didn’t like organization or health care provider
  10. Didn’t want to know, wasn’t ready
  11. Would make you change: how you thought about yourself, your whole life, etc.

1. Different tests may be right for different types of people. What kind of person would use _____________ test do you think?
2. Could you see yourself using different types of tests at different points in your life?
3. What is missing from available testing options that would make you test more regularly?

Now I am going to ask you some questions about your use of the internet and mobile applications (“apps”). We are not going to make a distinction between using a phone or a computer to access the internet or an app.

1. What do you use the Internet or apps for?
2. Where do you learn about new sites and/or apps?
3. What sites or apps do you visit/use at least once per day? Once per week?
4. What sites or apps do you like the most?
   1. What is appealing about these sites/apps?
      1. Function
      2. Look/visuals
      3. simplicity/complexity
   2. What don’t you like about these sites/apps?
5. Have you ever used the Internet or an app for a health-related reason? (for example, tracking your workouts, or blood pressure or blood sugar)
   1. What was that like for you? Did you enjoy it? Did it help you?
   2. Did using it last or did the appeal wear off? What kept you using it?

We are developing a web program/phone app to encourage HIV testing among young Black men/women who have sex with men. The intervention would be a brief and could be used on mobile phones or a computer. It will provide men/women with a personalized recommendation of ways to get an HIV test which takes into account barriers and preferences that they have about HIV testing.

1. What do you think about such a program/app? How might you use it?
   1. What are some of the positives or good things about such a program/app?
   2. What are some of the negatives or not so good things about such a program/app?
   3. Do you have any ideas on how to make it as user friendly and effective as possible?

Now, we will talk about some of your thoughts and opinions about community experiences related to HIV testing.

1. What are the messages coming out of the __________[FILL IN WITH SALIENT COMMUNITY HERE] related to HIV testing?
   1. How have you experienced this community talking about HIV testing?
   2. Where do you think these messages come from?
   3. What do you think about these messages? What have been some of your experiences related to these messages?
2. How have you experienced Black men/women like yourself talking about HIV testing?
   1. Can you share some of these experiences of what they are saying about HIV testing?
   2. Do you have any stories from your own experience that relate to these messages?

**Now, we will talk about experiences that you’ve had related to sex and relationships. We will focus on your most recent sexual experience.**

1. Tell me about the last time you had sex. How did it happen?

**Probes:**

- How did you meet?
- Where did you meet?
- What happened?
- Where did the sex occur?
- What kind of sex occurred?

1. How were you feeling emotionally at that time (earlier that day prior to the sexual experience)? For example, what kind of mood were you in?
2. What attracted you to this partner(s)? Was this your usual “type” of partner?
3. Were you planning on having sex?
4. Did you and your sexual partner(s) talk about sex before having sex? What kinds of things did you talk about? What were some of the things you thought about but didn’t talk about?
5. Did you talk about HIV or HIV testing?
   1. If so, how did it come up? Did it come up before or after you had sex?
      1. [If HIV was discussed] Did HIV testing come up in your conversation about HIV?
      2. Did knowing or not knowing your partner’s HIV status influence the kind of sex that you had together? If so, how?
   2. If not, did you feel like you did not need to discuss it? If not, did you want to discuss it? (Explore barriers to communication involving HIV status).

Now, we will discuss your thoughts about the prevention needs of young Black men/women who have sex with men.

1. What do you think you need to help you stay sexually healthy and/or prevent getting or giving HIV?
2. What do you think are the HIV prevention needs or things that would improve the lives of young Black men/women who have sex with men in your community? Where does HIV testing fit into this? What may be some of the things that would get in the way of making these changes?

We will be closing our interview in a few minutes. But, I would like to ask you a couple of more questions.

What was it like talking about some of your experiences during this interview?

Is there anything else that you would like to tell me that we haven’t talked about?

We very much appreciate your time and sharing your experiences during this interview. After I turn off the tape, we will discuss any referrals or resources that you might need. Once again, thank you for your time.

If participant is 16 or 17 years old: The advocate is available to meet with you at this time to answer any of your questions about the study.
